# Editorial: Advances in Spinal Cord Epidural Stimulation for Motor and Autonomic Functions Recovery After Severe Spinal Cord Injury

**DOI:** 10.3389/fnsys.2021.820913

**Published:** 2022-01-06

**Authors:** Enrico Rejc, Claudia A. Angeli, Ronaldo M. Ichiyama

**Affiliations:** ^1^Kentucky Spinal Cord Injury Research Center, University of Louisville, Louisville, KY, United States; ^2^Department of Neurological Surgery, University of Louisville, Louisville, KY, United States; ^3^Frazier Rehabilitation Institute, University of Louisville Health, Louisville, KY, United States; ^4^Department of Bioengineering, University of Louisville, Louisville, KY, United States; ^5^Faculty of Biological Sciences, School of Biomedical Sciences, University of Leeds, Leeds, United Kingdom

**Keywords:** spinal cord injury, motor function, autonomic function, bladder, locomotion, voluntary movement, cardiovascular, recovery

## Introduction

Spinal cord injury (SCI) disrupts the communication within the nervous system, leading to loss of sensorimotor function caudal to the level of injury and loss of autonomic function. Individuals with more complete and chronic injuries have poor prognosis for neurological and functional recovery, and suffer from a significant decrease in quality of life. To date, standard of care for individuals with chronic, severe SCI is primarily focused on implementing compensatory strategies and managing SCI-related health complications. However, in the last decade, clinical studies implementing epidural electrical stimulation of the lumbosacral spinal cord (scES) in individuals with complete paralysis from SCI resulted in the unprecedented proof of principle that recovery of motor function, even at a chronic stage, is potentially available (Harkema et al., 2011; Angeli et al., 2014, 2018; Rejc et al., 2015; Gill et al., 2018; Wagner et al., 2018). More recent evidence also suggested that scES has the potential to regulate autonomic functions in this population (Harkema et al., 2018; Herrity et al., 2018; Squair et al., 2021). While these scientific findings have brought hope for recovery of lost functions to millions of individuals worldwide living with a SCI, further efforts are needed to achieve an effective clinical translation of this spinal cord stimulation technology.

The present Research Topic includes original human- and rat-model studies and a “hypothesis and theory” article focused on the mechanisms underlying spinal cord electrical stimulation and its positive multi-system effects targeting motor, bladder, cardiovascular, and immune functions. Of the 18 manuscripts that were initially submitted, 13 were accepted for publication. A total of 89 authors contributed to this Research Topic, the content of which has been viewed over 80,000 times since its launch in August 2019. When considering these numbers, themes and content of the manuscripts included in the present Research Topic, we believe that it can provide important perspectives to the field of spinal cord neuromodulation in SCI. So, we would like to thank our authors, referees, and above all our readers for having supported this project. The Topic contributions are summarized below in two thematic areas: (i) motor function and (ii) autonomic function.

## Motor Function

To date, the prevailing view is that scES enhances motor function recovery after SCI by recruiting large dorsal root myelinated fibers associated with somatosensory information, and particularly proprioceptive information (Moraud et al., 2016), at their entry into the spinal cord as well as along the longitudinal portions of the fiber trajectories (Rattay et al., 2000; Capogrosso et al., 2013). This conceivably leads to altering the excitability of spinal circuits involved in motor patterns generation to a level that can enable sensory information and residual supraspinal inputs to become sources of motor control (Rejc and Angeli, 2019).

Militskova et al. proposed a structured approach that combines electrophysiological techniques with positional changes and participant-driven reinforcement maneuvers to assess the neurophysiological profile of individuals with clinically motor complete SCI, as well as the influence of weight bearing-related sensory information and supraspinal inputs on the excitability of the spinal circuitry caudal to the level of injury. This approach can be helpful to characterize the neurophysiological completeness of SCI, contributing to research participants' selection for upcoming scES clinical trials, and may also be implemented to assess the effects of activity-based training on neurophysiological profile. Gill M.L. et al. were also interested in investigating the effects of modulating afferent and supraspinal inputs on motor output in two individuals with motor complete SCI receiving scES. In particular, they demonstrated that the level of body weight support and the participant's intent to step on a treadmill modulated the stepping pattern facilitated by scES. In particular, lower body weight support (20% body weight) and the intent to step improved stepping independence and decreased clinician assistance. This research group (Gill M. et al.) also reported that scES improved aspects of sitting reaching performance in the same two SCI research participants, which is relevant to enhance the independent performance of activities of daily living. scES improved the forward reaching distance, while it had negligible effect on the lateral reach distances. Also, reach distances were larger when individuals were assessed from their wheelchair compared to sitting on a mat, emphasizing the importance of pelvic stabilization provided by the wheelchair cushion.

The recovery of volitional lower limb motor control promoted by scES in individuals with clinically motor complete SCI entails that residual supraspinal connectivity to the lumbosacral spinal circuitry still persists in these individuals.  Rejc et al. aimed at exploring further the mechanisms underlying scES-promoted recovery of volitional lower limb motor control by investigating neuroimaging markers at the spinal cord lesion site via magnetic resonance imaging. Amount and location of spared spinal cord tissue at the lesion site were not related to the ability to generate volitional leg movements prior to any training with scES. On the other hand, spared tissue of specific cord regions significantly and importantly correlated with inhibitory and coordination aspects of motor control. Peña Pino et al. presented evidence for recovery of volitional lower limb movement in the absence of spinal stimulation following long-term application of scES. In particular, four out of seven participants with a chronic, clinically motor complete SCI developed the ability to voluntarily move their lower extremities without stimulation. Interestingly, the sub-group that was successful in generating movement without stimulation presented higher spasticity scores prior to the beginning of scES. This may suggest that the volitional movements generated with scES over a long period of time could result in motor re-learning that takes advantage of spasticity by modulating it to achieve the targeted motor outcomes. Spasticity is also often present in individuals with cerebral palsy, largely reflecting a functionally abnormal spinal-supraspinal connectivity. Edgerton et al. proposed a model of spinal cord stimulation in cerebral palsy, hypothesizing that spinal neuromodulation in combination with proprioceptive-activity based training can transform the dysfunctional spinal-supraspinal connections improving function.

Another area covered in this Research Topic is related to novel strategies aimed at optimizing neuromodulation outcomes.  Taccola et al. explored the combination of scES and pharmacological interventions targeting adenosine A1 receptors as precursors to engage the sensorimotor networks and promote restoration of function in rats following chronic severe SCI. The different effects promoted by this combinatorial treatment in intact and injured animals support dedicated follow-up studies. Finally, Shkorbatova et al. presented a comparison of spinal stimulation methods that may be alternative to scES. In particular, a novel model of transvertebral stimulation was compared to transcutaneous spinal cord stimulation in rats, studying the muscle activation selectivity associated with these two neuromodulation approaches. Transvertebral stimulation had similar effects on the spinal sensorimotor networks compared to transcutaneous stimulation, and could be a viable neuromodulation strategy to be investigated in the future.

## Autonomic Function

Evidence to date suggests that spinal cord stimulation can target and regulate neural networks controlling multiple organ systems. Kreydin et al. evaluated the long-term effects of transcutaneous stimulation focused on bladder function in individuals with SCI, stroke, and multiple sclerosis. After the spinal cord stimulation period, positive outcomes including decreased detrusor overactivity, improved continence, and enhanced bladder sensation were found across the different subgroups. On the other hand, no changes in voiding efficiency were observed after the intervention when spinal stimulation was not applied. Herrity et al. provided evidence for improvements in bladder function following motor- and/or cardiovascular-specific scES and activity-based training in individuals with chronic, motor complete SCI. Bladder capacity without stimulation improved post-intervention, and this adaptation was retained at the 1-year follow-up. Detrusor pressure and bladder compliance also improved following intervention, but returned to baseline levels at follow-up. Although showing successful bladder outcomes, activity-based interventions with scES did not attenuate the increases in systolic blood pressure as a result of bladder distention; this suggests the need of bladder-specific scES parameters that also include a component for blood pressure modulation. To this end, Sysoev et al. evaluated the effects of scES sites on detrusor and external sphincter muscles following a thoracic (T)8 lateral hemisection in a rat model. Results indicated that the activation of the detrusor and external sphincter were optimal at different stimulation sites, supporting the need for specific stimulation configurations to address bladder dysfunction following SCI.

The effects of scES on other organ systems were assessed in pilot human studies.  Legg Ditterline et al. evaluated the cardiac structure and function adaptations following multiple scES interventions. Aortic root, left atrial and ventricular chamber dimensions and mass were all increased following scES interventions. These structural changes accompanied improved blood pressure regulation following scES interventions, and have implications for improved quality of life as well as reduction in cardiovascular morbidity. Finally, Bloom et al. evaluated the immune responses to long-term scES targeted to improve cardiovascular function. In a case study involving a chronic, cervical motor complete SCI individual, changes in whole-blood gene expression following the scES intervention suggested an improved immune system function. Inflammatory pathways were downregulated while adaptive immune pathways were upregulated. These findings provide additional evidence for holistic improvements associated with prolonged use of scES that might affect quality of life and reduce the burden of SCI in the affected individuals.

In conclusion, spinal cord neuromodulation in the form of epidural and transcutaneous electrical stimulation can provide a wide range of multi-system benefits for individuals with severe SCI and other neurological disorders. The exciting novel advances supporting recovery of motor, cardiovascular, and bladder functions by spinal cord electrical neuromodulation can provide real-world benefits to individuals that suffer from SCI. In the near future, parallel efforts by the scientific and clinical community to translate spinal cord electrical stimulation approaches to larger clinical trials, and to further improve their efficacy (i.e., by improved stimulation technology and/or combinatorial treatments) are warranted.

## Author Contributions

ER and CA wrote the first draft of the manuscript. All authors contributed to the conception of the manuscript, revised the manuscript, and approved its final version.

## Funding

ER and CA were supported by Christopher & Dana Reeve Foundation, Kessler Foundation, the Leona M. and Harry B. Helmsley Charitable Trust, and Craig H. Nielsen Foundation. RI was supported by the International Spinal Research Trust (NMN007 and BBS003), and by Brain Research UK (WMCR P73576).

## Conflict of Interest

The authors declare that the research was conducted in the absence of any commercial or financial relationships that could be construed as a potential conflict of interest.

## Publisher's Note

All claims expressed in this article are solely those of the authors and do not necessarily represent those of their affiliated organizations, or those of the publisher, the editors and the reviewers. Any product that may be evaluated in this article, or claim that may be made by its manufacturer, is not guaranteed or endorsed by the publisher.
